# Supplementary material for: Examining the potential impacts of a coastal renourishment project on the presence and abundance of Escherichia coli
Source: PLoS One. 2024 May 24;19(5):e0304061. doi: 10.1371/journal.pone.0304061 (PMC11125542; doi:10.1371/journal.pone.0304061)
Supplement: S1 Table — Summary of E. coli Two-Way ANOVAs with corresponding F-ratios and P values. (PDF) [file pone.0304061.s001.pdf]

**Supporting Information File 1**: Summary of *E. coli* Two-Way ANOVAs with corresponding F-ratios and P values.

| **Two-Way ANOVA Summary** | **F- Ratio** | **P-Value** |
|---|---|---|
| Whole Model Summary | **9.1440** | **<0.0001** |
| Effect of Sub-Section 2014 | **17.325** | **<0.0001** |
| Effect of Renourishment 2014 | 2.8908 | 0.0928 |
| Interactive Effect 2014 | **4.312** | **0.0165** |
| Whole Model Summary | 1.349 | 0.2517 |
| Effect of Sub-Section 2015 | 0.550 | 0.5791 |
| Effect of Renourishment 2015 | **4.137** | **0.0408** |
| Interactive Effect 2015 | 0.465 | 0.6297 |
