# Supplementary material for: Examining the potential impacts of a coastal renourishment project on the presence and abundance of Escherichia coli
Source: PLoS One. 2024 May 24;19(5):e0304061. doi: 10.1371/journal.pone.0304061 (PMC11125542; doi:10.1371/journal.pone.0304061)
Supplement: S2 Table — Summary of ANOVA Results for Sand Grain Size Analysis with F and P values. (PDF) [file pone.0304061.s002.pdf]

Supporting Information File 2: Summary of ANOVA Results for Sand Grain Size Analysis with F and P values.

Year	Category	Source	F-Ratio	P- Value
2014	Very Coarse	Renourishment Status	0.5341	0.4670
		**Beach Zone**	**5.6972**	**0.0048**
		Interaction	2.1205	0.1265
	Coarse	Renourishment Status	3.1112	0.0815
		**Beach Zone**	**3.9014**	**0.0241**
		Interaction	5.3040	0.0068
	Medium	Renourishment Status	0.9642	0.3290
		**Beach Zone**	**4.8916**	**0.0098**
		Interaction	0.1787	0.8367
	Fine	Renourishment Status	2.3534	0.1289
		**Beach Zone**	**4.5221**	**0.0137**
		Interaction	1.8569	0.1627
	**Very Fine**	**Renourishment Status**	**39.1752**	**<.0001**
		**Beach Zone**	**14.7481**	**<.0001**
		**Interaction**	**7.7007**	**0.0009**
2015	Very Coarse	Renourishment Status	0.1817	0.1817
		**Beach Zone**	**9.1014**	**0.0003**
		Interaction	1.5504	0.2189
	Coarse	**Renourishment Status**	**17.9049**	**<.0001**
		Beach Zone	0.1338	0.8749
		**Interaction**	**4.3277**	**0.0164**
	Medium	Renourishment Status	0.7302	0.3954
		**Beach Zone**	**17.7440**	**<.0001**
		Interaction	1.9315	0.1516
	Fine	**Renourishment Status**	**15.7223**	**0.0002**
		Beach Zone	0.7385	0.4810
		Interaction	2.8622	0.0630
	**Very Fine**	**Renourishment Status**	**25.8260**	**<.0001**
		**Beach Zone**	**3.8613**	**0.0251**
		**Interaction**	**6.5285**	**0.0024**
