# Supplementary material for: Examining the potential impacts of a coastal renourishment project on the presence and abundance of Escherichia coli
Source: PLoS One. 2024 May 24;19(5):e0304061. doi: 10.1371/journal.pone.0304061 (PMC11125542; doi:10.1371/journal.pone.0304061)
Supplement: S3 Table — Summary of sand grain analyses for 2014 samples. The number represents the percentage of the total sand sample belonging to each grain size category. P-values in bold symbolize statistically significant results in the t-tests between the sections. (PDF) [file pone.0304061.s003.pdf]

Supporting Information File 3: Summary of sand grain analyses for 2014 samples. Number represents the percentage of the total sand sample belonging to each grain size category. P-values in bold symbolize statistically significant results in the t-tests between the sections.

Grain Category	Beach Sub-Section	Non-nourished Mean%	Renourished Mean %	t- ratio	P-Value
Very Coarse	Dune	0.251427	2.831388	1.1667	0.2467
Coarse	Dune	3.41045	4.26953	0.1950	0.8459
Medium	Dune	37.78838	35.15669	0.4269	0.6705
Fine	Dune	54.29176	50.10464	0.6899	0.4922
**Very Fine**	**Dune**	**2.60012**	**6.26466**	**3.7629**	**0.0003**
**Very Coarse**	**Intertidal**	**1.12265**	**6.02131**	**2.2153**	**0.0295**
**Coarse**	**Intertidal**	**2.40758**	**14.99676**	**2.8572**	**0.0054**
Medium	Intertidal	46.88611	39.27975	1.2340	0.2207
Fine	Intertidal	42.26409	36.50288	0.9494	0.3452
Very Fine	Intertidal	1.31839	2.13611	0.8397	0.4035
Very Coarse	Subtidal	7.876852	6.131349	0.7308	0.4670
Coarse	Subtidal	17.05730	8.66291	1.7639	0.0815
Medium	Subtidal	32.16280	25.62553	0.9819	0.3290
Fine	Subtidal	39.37203	49.42761	1.5341	0.1289
**Very Fine**	**Subtidal**	**2.21236**	**8.52954**	**6.2590**	**<.0001**
