# Supplementary material for: Examining the potential impacts of a coastal renourishment project on the presence and abundance of Escherichia coli
Source: PLoS One. 2024 May 24;19(5):e0304061. doi: 10.1371/journal.pone.0304061 (PMC11125542; doi:10.1371/journal.pone.0304061)
Supplement: S4 Table — Summary of sand grain analyses for 2015 samples. The number represents the percentage of the total sand sample belonging to each grain size category. P-values in bold symbolize statistically significant results in the t-tests between the sections. (PDF) [file pone.0304061.s004.pdf]

Supporting Information File 4: Summary of sand grain analyses for 2015 samples. Number represents the percentage of the total sand sample belonging to each grain size category. P-values in bold symbolize statistically significant results in the t-tests between the sections.

Grain Category	Beach Sub-Section	Non-nourished Mean%	Renourished Mean %	t- ratio	P-Value
Very Coarse	Dune	1.08550	3.84379	0.8603	0.3924
Coarse	Dune	5.32029	5.14378	0.0915	0.9274
Medium	Dune	72.87759	64.64381	0.8855	0.3786
Fine	Dune	13.49660	21.19777	1.1772	0.2426
Very Fine	Dune	1.39245	3.40113	1.7017	0.0927
Very Coarse	Intertidal	1.13649	3.72375	0.8069	0.4223
**Coarse**	**Intertidal**	**3.44438**	**7.35194**	**2.0247**	**0.0462**
Medium	Intertidal	80.51045	63.44054	1.8357	0.0701
Fine	Intertidal	9.90847	14.20430	0.6567	0.5133
Very Fine	Intertidal	1.75315	1.58419	0.1431	0.8865
Very Coarse	Subtidal	14.52933	9.36292	1.3481	0.1817
**Coarse**	**Subtidal**	**2.10013**	**9.66064**	**4.2314**	**<.0001**
Medium	Subtidal	34.53268	41.88914	0.8545	0.3954
**Fine**	**Subtidal**	**4.25999**	**28.27502**	**3.9651**	**0.0002**
**Very Fine**	**Subtidal**	**1.07001**	**6.62360**	**5.0819**	**<.0001**
